# Effects of social sustainability signaling on neural valuation signals and taste-experience of food products

**DOI:** 10.3389/fnbeh.2015.00247

**Published:** 2015-09-08

**Authors:** Laura Enax, Vanessa Krapp, Alexandra Piehl, Bernd Weber

**Affiliations:** ^1^Department of Epileptology, University Hospital BonnBonn, Germany; ^2^Department of NeuroCognition/Imaging, Life and Brain CenterBonn, Germany; ^3^Center for Economics and Neuroscience, University of BonnBonn, Germany

**Keywords:** functional magnetic resonance imaging, food choice, food labels, ventral striatum, vmPFC, sustainability, Fair Trade

## Abstract

Value-based decision making occurs when individuals choose between different alternatives and place a value on each alternative and its attributes. Marketing actions frequently manipulate product attributes, by adding, e.g., health claims on the packaging. A previous imaging study found that an emblem for organic products increased willingness to pay (WTP) and activity in the ventral striatum (VS). The current study investigated neural and behavioral processes underlying the influence of Fair Trade (FT) labeling on food valuation and choice. Sustainability is an important product attribute for many consumers, with FT signals being one way to highlight ethically sustainable production. Forty participants valuated products in combination with an FT emblem or no emblem and stated their WTP in a bidding task while in an MRI scanner. After that, participants tasted—objectively identical—chocolates, presented either as “FT” or as “conventionally produced”. In the fMRI task, WTP was significantly higher for FT products. FT labeling increased activity in regions important for reward-processing and salience, that is, in the VS, anterior and posterior cingulate, as well as superior frontal gyrus. Subjective value, that is, WTP was correlated with activity in the ventromedial prefrontal cortex (vmPFC). We find that the anterior cingulate, VS and superior frontal gyrus exhibit task-related increases in functional connectivity to the vmPFC when an FT product was evaluated. Effective connectivity analyses revealed a highly probable directed modulation of the vmPFC by those three regions, suggesting a network which alters valuation processes. We also found a significant taste-placebo effect, with higher experienced taste pleasantness and intensity for FT labeled chocolates. Our results reveal a possible neural mechanism underlying valuation processes of certified food products. The results are important in light of understanding current marketing trends as well as designing future interventions that aim at positively influencing food choice.

## Introduction

Value-based decision making occurs when individuals choose between complex alternatives based on subjective values placed on the options and their attributes (Rangel et al., 2008). This computational model can also be applied to food decisions (Rangel, 2013). Rangel suggested that a food item is mapped into different attributes and a value is assigned to each of the attributes based on their individual contribution to rewards (e.g., based on physiological needs and prior experiences). Critically, attributes can be divided into (1) attributes associated with basic and immediate outcomes, such as taste, which are taken into account by all people, as well as (2) more abstract attributes, such as long-term health consequences, which are taken into account only by some people. Based on those attribute values, an overall item value is computed (Rangel, 2013). In a consumer context, choices are not only influenced by the available options, but also by contextual marketing cues, such as prices (Plassmann et al., 2008), brands (Philiastides and Ratcliff, 2013), or nutrition labels (Enax et al., 2015). A Fair Trade (FT) label on consumer products conveys information on social standards during the production process. Also, the label may convey inherently rewarding properties (e.g., feeling good about oneself for doing something good). These attributes could be regarded as rather abstract product attributes. One could argue that the FT emblem leads to attribute awareness (i.e., most decision-makers take into account this attribute and assign a value to it), subsequently increased weight on this attribute, and possibly a higher valuation, e.g., higher willingness to pay. However, it remains unclear, which brain regions are responsible for mediating the valuation bias toward FT products. The cortico-ventral basal ganglia circuit, which includes the ventromedial prefrontal cortex (vmPFC), anterior cingulate cortex (ACC), and the ventral striatum (VS), is important for reward processing and subsequent decision-making (Haber, 2011). The VS processes sensory, emotional and motivational information that drives action selection, action output and learning (Everitt and Robbins, 2005; Volkow et al., 2006; Porrino et al., 2007; Haber, 2011). A recent neuroimaging study found that an emblem for organic products increased food valuation and choice, both behaviorally and neurally (Linder et al., 2010). Specifically, an organic emblem increased activation in the VS, suggesting that the organic emblem conveys positive information that activates the dopaminergic reward system (O'Doherty et al., 2002; Beaver et al., 2006; Linder et al., 2010; Haber, 2011). The FT emblem, similar to an organic emblem, is expected to convey positive, possibly rewarding information about an item. The VS is part of the dopaminergic reward system, which modulates the hedonic element of learning, i.e., adapting behavior dependent on past rewarding or non-rewarding experience (Arias-Carrión et al., 2010). In addition to the connotations associated with organic products, the FT emblem also conveys information on ethical and moral standards. Several studies have demonstrated the importance of medial prefrontal regions and the PCC for processing stimuli with moral content or moral judgments (Greene et al., 2001, 2004; Raine and Yang, 2006). Product labels, that is, abstract attributes on food products, may also be more salient, compared to non-labeled alternatives. Regions from the “saliency” and attention network may therefore mediate the effect of labeling on behavior. The bilateral anterior insula and the ACC were proposed to form a saliency network (Seeley et al., 2007). The authors proposed that the ACC integrates sensory, autonomic and hedonic information and then adjusts behavior, as it is highly connected to subcortical and limbic structures (Seeley et al., 2007). Also, the VS was shown to be activated due to the saliency of stimuli, independent of the stimuli's rewarding properties (Zink et al., 2003). Critically, reward and attention are closely linked, and most studies cannot clearly distinguish between either of them. For example, one can expect that subjects will allocate more attention to more rewarding stimuli (Maunsell, 2004). As it was shown in a previous study on organic product labeling (Linder et al., 2010), we expect increased striatal activity. However, it is important to better characterize the observed striatal activity. If the striatal activity is due to salience only, this region should not correlate with the subjective value of a product, that is, WTP, or the increment value for FT products.

Another question that we want to address in this study is how the value computations are influenced by product labels, that is, we want to better characterize the neural network that alters the subjective value of products. Previous studies showed that consumers' ethical concerns are important drivers of food selection and consumption, and the willingness to purchase products produced according to ethical standards, such as FT labeled products, is rising (Honkanen et al., 2006; de Ferran and Grunert, 2007). The vmPFC has been shown to be important for value computations, as activity in the vmPFC is robustly correlated with subjective values, e.g., the WTP for consumer products (Plassmann et al., 2007; Bartra et al., 2013; Clithero and Rangel, 2014). In addition to computations of immediate values, the vmPFC was shown to also be involved in encoding expected values of outcomes (Plassmann et al., 2010; Noonan et al., 2011). A previous study showed that action-specific value information can be found in the vmPFC, suggesting that this region is an important mediator between reward and adaptive decision-making (FitzGerald et al., 2012). Also, the vmPFC was shown to correlate with the value of a chosen stimulus before taking into account the actions required to obtain the different stimuli, suggesting that stimulus values are directly compared to make choices, and that the vmPFC plays a crucial role in this process (Wunderlich et al., 2010). Beliefs and expectations can alter the value signal in the vmPFC (McClure et al., 2004; de Araujo et al., 2005; Plassmann et al., 2008). Different attributes of a stimulus that are likely represented outside of the vmPFC need to be integrated to construct a valuation signal (Smith et al., 2014). In addition to the role of the vmPFC in the encoding of general stimulus values, it also has a role as a decision value comparator in multi-alternative choices. The vmPFC was shown to integrate various sources of evidence encoded in different brain areas, and does so by using comparator operations (Philiastides et al., 2010). In other words, value signals in the vmPFC take into account different types of attributes and their respective weights (Rangel, 2013). For example, the vmPFC value signal in healthy eaters weighs both taste and health attributes of foods (Hare et al., 2009). This implies that the vmPFC needs to be densely connected with various brain regions, such as other reward-related areas as well as attention-related and higher cognitive areas. For example, vmPFC and VS are structurally and functionally connected (Hedreen and Delong, 1991; Haber et al., 1995; Goldstein and Volkow, 2002; Haber, 2011; Yu et al., 2013). Connections between vmPFC and the striatum were shown to be important for reward-based learning (Haber et al., 2006). Studies dealing with addiction suggest that vmPFC-striatal glutamatergic projections corroborate the transmission from the subjective value of the reinforcer, which is represented in the vmPFC, to craving sensations, which are generated in the striatum (Goldstein and Volkow, 2002; Kalivas and Volkow, 2005; Yu et al., 2013). It was shown that outcome evaluations enter the corticostriatal pathway through the vmPFC and then project to the VS, possibly to adapt striatal activity based on the value computed in the vmPFC (Yu et al., 2013). Also, the VS projects back to the vmPFC, possibly to update the hedonic responses or experienced value (Haber and Knutson, 2010; Yu et al., 2013). Further, other regions of interest that may mediate the bias toward labeled products include regions from the saliency network, such as the ACC. The ACC was also shown to be connected to the vmPFC and VS during decision-making (Cohen et al., 2005). Also, the PCC and vmPFC were shown to be densely connected, both structurally and functionally (Greicius et al., 2009; Leech and Sharp, 2014).

Marketing cues can alter consumption experiences and have been consistently shown to induce so called “placebo effects,” i.e., modifying contextual components of a product can alter experienced pleasantness and efficacy of identically composed products (Shiv et al., 2005; Grabenhorst et al., 2008; Plassmann et al., 2008; Plassmann and Weber, 2015). For instance, taste perception was shown to be affected by knowledge about a product's brand, its ingredients or packaging (Allison and Uhl, 1964; Lee et al., 2006). Prices can also serve as an external cue generating expectations about the product's quality (Plassmann et al., 2008). A previous study found that a food's ethicality and the resulting moral satisfaction, although mostly unrelated to a product's quality, influenced subjective taste perception. Further, they suggested that the augmented taste experience of ethical labeled foods also reinforced further purchases of these food products (Bratanova et al., 2015). Critically, not only self-reported, but also neural measures of consumption enjoyment can be altered by cognitive concepts, such as pricing (Plassmann et al., 2008), or verbal descriptions (de Araujo et al., 2005). A more detailed understanding of brain processes underlying valuation and expectancy during choice and consumption of “certified” food products is crucial to further understand current marketing trends as well as to design future interventions that aim at positively influencing food choice (Plassmann and Weber, 2015).

Here, we investigate the influence of an FT emblem on food valuation and choice using an fMRI paradigm and a subsequent taste experiment. Inside the scanner, subjects saw and bid on different food products presented either with an FT emblem or without an emblem. In a further behavioral task, participants tasted and rated chocolates labeled with either an FT emblem or marked as “conventionally produced.” Unbeknown to the participants, both chocolates were identical.

We hypothesize that FT labeled food products are evaluated more positively compared to conventionally produced products. This can be seen on the behavioral level via increased WTP in the bidding task and increased reported taste experience in a taste rating task of identical chocolates. On the neural level, we expect increased activity in reward-related regions, such as the VS, and also in attention- and saliency-related areas, such as the ACC and anterior insula. As shown in previous studies (Plassmann et al., 2007; Hare et al., 2009, 2011; Rangel, 2013; Clithero and Rangel, 2014), we expect that the vmPFC correlates with the subjective value of the products. The vmPFC is thought to integrate various attributes into a common valuation signal (Hare et al., 2011; Rangel, 2013). In order to integrate input into a common value signal, we expect increased connectivity between the vmPFC and regions important for saliency and reward, such as the VS and ACC, for FT labeled products.

## Materials and methods

### Participants

Forty participants (21 females, 3 left-handed) between 19 and 33 years [mean (*M*) = 24.08, standard deviation (*SD*) = 3.60] participated in this study. Standard exclusion criteria for MRI studies were applied. The study was approved by the local ethics committee and all participants provided written informed consent. For the fMRI analysis, a total of 33 subjects were used: of the 40 subjects four participants were excluded due to excess translational and rotational head movement (>3 mm and >2.5°, respectively), two were excluded due to technical problems and one due to a lesion in the MRI scan.

### Stimuli

Color images of 40 food products (e.g., chocolate, coffee, rice) were presented on a black background via video goggles (Nordic NeuroLab, Bergen, Norway) at a resolution of 800 × 600 pixels. The design was presented using Presentation©software (NeuroBehavioral Systems Inc.). Products were selected based on their availability in both FT and conventional forms. Brand-related information was removed from the product images. Subjects saw each product twice—once with and once without the FT emblem. The sequence of the stimuli was randomized with the constraint that the two conditions of the same product were not displayed directly one after another. For the chocolate tasting, a conventional chocolate (Choceur Alpenmilch, Aldi South) was used. Two pieces of this chocolate were each quartered for every participant and then equally distributed on two small plates. One plate was served as the FT chocolate and the other as the conventional one. Prior to the task, subjects were informed that products shown with an FT emblem were produced according to FT standards.

### fMRI task

**Figure 1:**
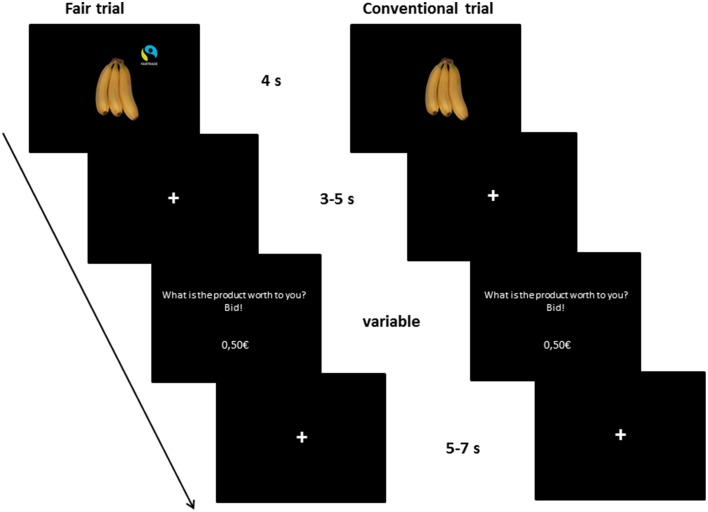
**Illustration of the trial setup**. The fMRI task consisted of 80 trials. Participants saw 40 different products twice, once labeled with a Fair Trade emblem, once without a label. The product was displayed for 4 s, followed by a fixation (3–5 s). Then, subjects were prompted to enter the amount of money they were willing to pay for the presented product in Euro (€). They could enter the price with a precision of five cent using four buttons (1 = +50 cent, 2 = −10 cent, 3 = +5 cent, 4 = confirm the bid). The price was updated after each button press. The duration for entering the WTP was dependent on the subjects' individual speed. Afterwards, a fixation cross was shown (5–7 s). The order of the buttons was counterbalanced across subjects.

Participants started with a short practice session on a computer to familiarize themselves with the task. In this practice session, subjects had to enter four given prices correctly and then had to bid on four products as in the following fMRI task. The fMRI experiment consisted of 80 trials, see Figure 1 for an overview. At the beginning of each trial, a product was displayed for 4 s, followed by a fixation (3–5 s). Then, subjects were prompted to enter the amount of money they were willing to pay for the presented product. They could enter the price with a precision of five cent using four buttons (1 = +50 cent, 2 = −10 cent, 3 = +5 cent, 4 = confirm the bid). The price was updated after each button press. The duration for entering the WTP was dependent on the subjects' individual speed. Afterwards, a fixation cross was shown (5–7 s). The order of the buttons was counterbalanced across subjects. Subjects received €25 endowment for participation, which they could use for purchasing products. The Becker-DeGroot-Marschak auction was used as a widely-used model for market transactions in the laboratory in order to measure individual preferences and the exact WTP from each subject for every product (Becker et al., 1964; Plassmann et al., 2007). Three products were randomly chosen for implementation in the auction (see detailed procedure in Enax et al., 2015).

### Behavioral tasting task

After the fMRI task, subjects participated in a chocolate tasting. Data from all participants that completed the tasting and filled out the questionnaire were used (*N* = 38). First, participants had to state their expectancy on taste intensity and taste pleasantness of FT or conventional chocolates, respectively. Next, they tasted the respective chocolate and stated their experienced taste intensity and taste pleasantness. They then received water to rinse their mouth and the same procedure was repeated for the other condition. Unbeknown to the subjects, both tasted chocolates were identical. The conditions were randomized across subjects. A discrete Likert scale from 1 (not at all) to 7 (very much) was used for both ratings.

### Behavioral data analysis

Behavioral data were analyzed using R Studio (RStudio: Integrated development environment for R, Version 0.97.551, Boston, MA, USA). Bids for conventional and FT products (of the 33 participants included in the fMRI analyses) were analyzed using a linear-mixed model. We used a likelihood ratio test to obtain *p*-values by comparing the likelihood of the full model with label as a predictor and the model without the label as a predictor (null model). In detail, the models were set up as follows: The null model included a dummy for the fixed effect, subject as random effect and WTP as dependent variable. The full model included also product category (FT vs. conventional) as a fixed effect, plus a dummy fixed effect, subject as random effect and WTP as dependent variable. Model parameters were estimated using full maximum likelihood, which is necessary for model comparison using likelihood ratio tests (Pinheiro and Bates, 2000; Bolker et al., 2009; Winter, 2013). We compared the full model (with the fixed effect, label) against the reduced model; we conclude that the fixed effect is significant if the difference between the likelihood of those two models is significant (Winter, 2013). The models were compared with a Chi-Square test applied according to Wilk's Theorem stating that −2 times the log likelihood ratio of two models approach a Chi-Square distribution with degrees of freedom of the number of parameters that differ between both models (Winter, 2013), in this case, one parameter differs, namely product category, rendering one degree of freedom. Ratings in the chocolate-tasting task for all participants were analyzed using the Wilcoxon Signed-Ranks test and effect sizes *r* were calculated by $r=|Z\sqrt{N}|$ with *N* being the number of subjects used in the analysis and Z the respective *Z*-value of the test statistic. Further, correlations between expected and experienced taste intensity and pleasantness were calculated (Kendall's Tau correlation coefficient for non-parametric data).

### Image acquisition and preprocessing

MRI scanning was performed on a 1.5-Tesla Avanto scanner (Siemens, Erlangen, Germany), equipped with a standard eight-channel head coil. Functional scans were acquired using an echo-planar imaging (EPI) pulse sequence with repetition time (TR) = 2.5 s, echo time (TE) = 45 ms, and flip angle = 90°. Slice thickness was 3 mm with a slice gap of 0.3 mm. One volume consisted of 31 slices. Slices were acquired in an ascending manner and were axially oriented along the AC-PC plane with a tilt of −30° to optimize the signal in the OFC (Deichmann et al., 2003). The field of view (FOV) was 192 mm and the matrix size was 64 × 64. Additionally, a T1-weighted structural image was acquired at a resolution of 1 × 1 × 1 mm (TR = 1660 ms, TE = 3.09 ms, FOV = 256 mm). EPI images were motion corrected and realigned to the middle image of the time series. The mean realigned EPI image was segmented using six tissue probability maps and normalized to Montreal Neurological Institute (MNI) standard space. All other EPI images were co-registered to this normalized mean EPI image (resampled with 3 × 3 × 3.3 mm). Afterwards, smoothing was performed with a Gaussian kernel of 8 mm full width at half maximum. Preprocessed data were quality checked and then analyzed using SPM8, which was run with MATLAB 7.10.0 (R2010a, The Mathworks Inc, Natick, Massachusetts).

### First-level single-subject analysis

For statistical analyses, preprocessed data were corrected for non-sphericity using an autoregressive model and high-pass temporal filtered using a filter width of 128 s. A general linear model (GLM) was estimated for each participant. All regressors were convolved with a hemodynamic response function (HRF) and its time derivatives to consider for the hemodynamic response of the measured blood oxygenation level dependent signal. Each GLM included the bidding screen (during which subjects entered WTP via button presses), a constant session term and six covariates to capture residual movement-related artifacts as regressors of no interest. In GLM 1, the presentation of an FT product (“fair”) and the presentation of a conventional product (“conv”) were modeled. To investigate the main effect of the FT emblem, picture presentations were contrasted (fair vs. conv). GLM 2 was calculated to (1) identify brain regions in which activation correlates with WTP and (2) to investigate the effect of FT labeling over and above what is already conferred via WTP. For this purpose, we used two modulations for the onset of all pictures, that is, individual bids as parametric modulators for the product presentation and second, we used a dummy regressor (0 for conventional and 1 for FT products) as categorical modulator to investigate the effect of labeling beyond the WTP effect. We then calculated single-subject contrasts for all pictures modulated by WTP vs. baseline, as well as all pictures modulated by label vs. baseline. Due to the absence of a control image, we want to ascertain that the increased striatal activity is not merely due to increased salience (Zink et al., 2003) that arises as a result of the additional visual input (i.e., the FT logo). We therefore performed an additional analysis to ascertain that part of the striatum also correlates with subjective value (differences). Such a correlation would not be expected if this region is activated in response to salience only, as we hold the salience of the FT emblem constant across trials. As the same products were shown in both conditions, we tested whether brain activity in response to the product labeled with the FT logo is correlated with the increment value of the FT vs. conventional products at the time of FT product presentation. If the activity in the ventral striatum (and other brain regions) is only due to salience, this region should not correlate with the value difference. For each product *j* (*j* = 1–40) the increment value *i* of the FT vs. conventional products was calculated by subtracting the individual WTP for the conventional product *C*_*j*_ from the individual WTP for the same product labeled with an FT signal *F*_*j*_. We then performed an additional GLM (GLM 3) with the regressors FT product onset, and FT products modulated by *i* as well as onset of all conventional products, bidding period and movement regressors as regressors of no interest. First-level single-subject contrasts for FT modulated by *i* vs. baseline were calculated.

### Second-level group analysis

For the whole-brain analysis, first-level contrast images for each subject were entered into one-sample *t*-tests, treating subjects as a random variable. We performed whole-brain corrections for multiple comparisons at the cluster level (cluster-corrected threshold of *p* < 0.05). The voxel-level inclusion threshold was set to *p* < 0.005 with a minimal cluster extent of k ≥ 10 voxel (Lieberman and Cunningham, 2009). Brain activations were anatomically labeled according to the automated anatomic labeling tool implemented in bspmview and are reported using MNI coordinates.

### Region of interest analyses

We used the vmPFC as a region of interest that is expected to correlate with WTP. We used a 10 mm sphere around the peak voxel correlating with subjective value reported in a meta-analysis (*x* = 2, *y* = 46, *z* = −8, Bartra et al., 2013) for small volume (SV) correction analyses. A second region of interest is the left VS, due to the fact that it correlates with subjective value (Bartra et al., 2013) and showed robust activation in a prior organic labeling study (Linder et al., 2010). Also, it is part of the reward circuitry (Beaver et al., 2006; Haber, 2011) and usually does not survive cluster-wise FWE correction due to its small size. We used the peak coordinates reported in the same meta-analyses by Bartra and colleagues (*x* = −12, *y* = 12, *z* = −6).

### Psychophysiological interaction

Psychophysiological interaction (PPI) analyses were used as a method for investigating task-specific (i.e., label-specific in our case) changes in brain connectivity (Friston et al., 1997; O'Reilly et al., 2012). In order to analyze the network underlying valuation differences for FT vs. conventional products, we were interested in task-related functional connectivity of the regions activated in the main contrast fair > conv (GLM 1) to the vmPFC or possibly the left VS. First, we extracted the volumes of interest (VOIs) based on the peaks of the fair > conv contrast. VOIs were extracted using the SPM 8 Eigenvariate toolbox. We extracted each participant's principal eigenvariate around the individual-specific local maxima activation nearest to the peak voxel of the second level group analyses. The radius of the VOI spheres was 8 mm, and the search radios for local maxima from the group analyses was restricted to 16 mm for all regions, except for the ventral striatum, where we limited the search to 12 mm due to its smaller size. All voxels were significant at *p* < 0.1 uncorrected and the time series were adjusted for effects of interest. Variance associated with the six motion regressors was removed from the extracted time-series. We could not extract VOIs from four participants, as they did not show any suprathreshold activity in one or more of the VOIs. These participants were therefore excluded from the subsequent PPI analysis. The time courses were then deconvolved based on the model for the canonical hemodynamic response following the procedure proposed by Gitelman et al. (2003). On the single subject level, GLMs with the following regressors were modeled: (1) the PPI regressor, representing the interaction between label and neural activity in the seed VOIs (psychophysiological interaction variable); (2) the psychological regressor, representing an indicator for the main effect (i.e., fair > conv); and (3) the physiological regressor, namely the original BOLD zero-centered eigenvariate from the VOIs (the VOI time course). The first two regressors were forward-convolved with the canonical HRF and entered into the regression model. The model also included motion parameters as regressors of no interest. Single subject contrasts were calculated following estimation of the GLM, which were then submitted to a one-sample *t*-test.

### Dynamic causal modeling

Further, we investigated the effective connectivity between the regions that show a significant PPI effect (ACC, SFG, and VS) and the vmPFC. We used a relatively novel *post-hoc* dynamic causal modeling (DCM) procedure (Rosa et al., 2012). Please see the Supplementary Material for details.

## Results

### Bids in fMRI task

**Figure 2:**
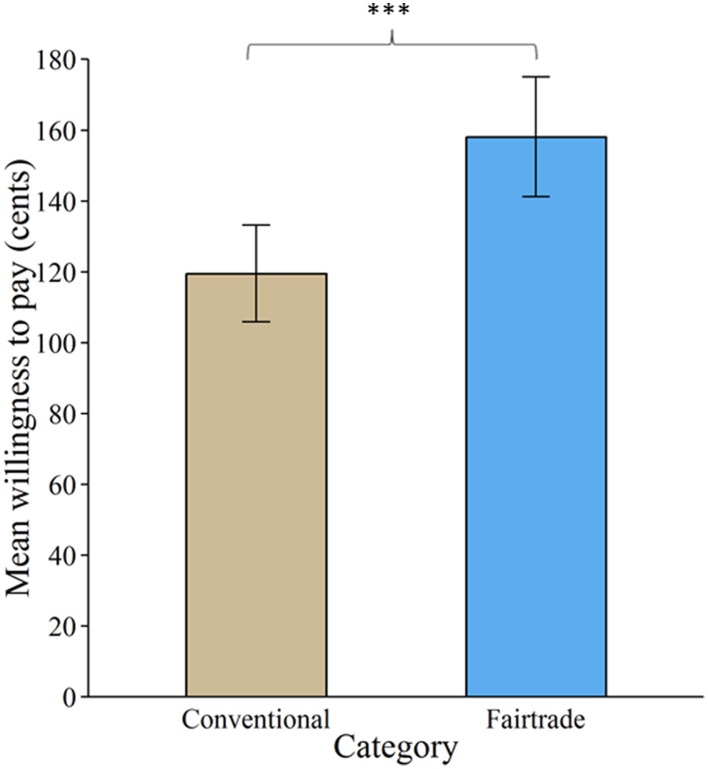
**Valuation of products labeled with a Fair Trade emblem vs. no label**. Willingness to pay increased significantly for products labeled with a Fair Trade emblem. Error bars indicate standard error of the mean. ^***^p < 0.001.

Overall, 97.54% of all bids were higher than zero. Product category significantly affected subjects' WTP [$\chi_{\left(1\right)}^{2}$ = 193.06, *p* < 0.001]. FT labeling increased WTP by about 38.6 cents (± 2.7 standard errors), see Figure 2.

### fMRI task

#### Main effects and parametric analysis

**Figure 3:**
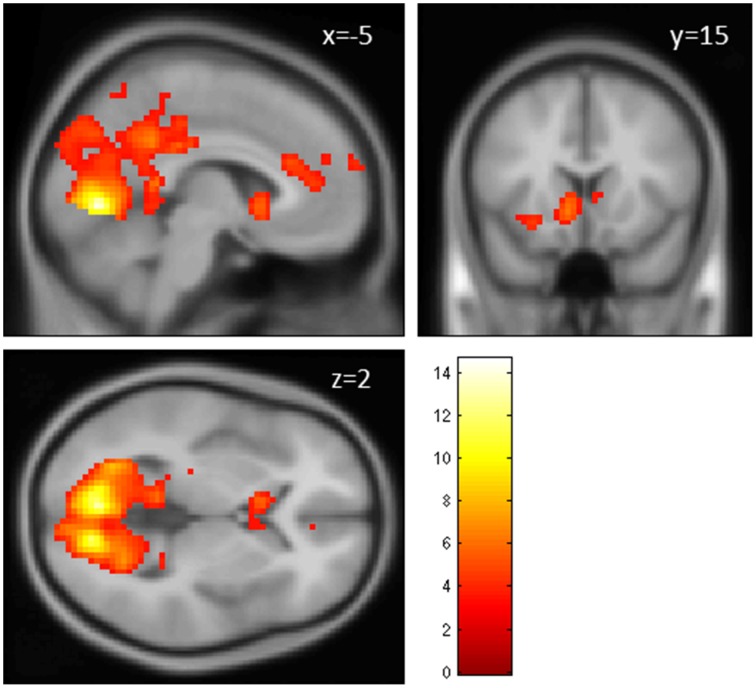
**Main effect of perceiving a Fair Trade emblem in conjunction with a food product vs. no emblem**. Activity increased in the ventral striatum, anterior cingulate cortex and occipital lobe at the time of evaluation (i.e., stimulus onset), displayed at *p* < 0.001, uncorrected, on the mean 1.5T structural SPM template. Color bars indicate *T*-values.

The contrast fair > conv (GLM 1) revealed increased activation in VS, ACC, superior frontal gyrus (SFG, part of the frontal pole/BA 10), occipital regions and PCC (*p* < 0.05, cluster-level FWE corrected, see Figure 3 and Table 1). The reverse contrast (conv > fair) revealed no supra-threshold activations.

**Table 1:** **Main effect Fair Trade emblem (GLM 1)**.

**Region name**	**Extent**	***t*-value**	***X***	***y***	***z***
L Lingual gyrus	6056	14.31	−15	−73	−5
R Lingual gyrus	6056	11.24	12	−79	1
R Cuneus	6056	8.25	9	−85	22
L ACC	539	6.03	0	35	13
L Superior frontal gyrus	539	4.48	−9	68	19
L Middle frontal gyrus	539	3.66	−36	50	16
L Caudate nucleus		5.84	−9	14	−5
L Temporal pole	318	4.33	−42	14	−20
L Insula lobe	318	3.25	−42	11	7

*Whole brain activation for the main contrast Fair vs. conventional at p < 0.005. Only clusters that survive cluster-wise FWE correction (p < 0.05) are displayed. Brain regions are labeled according to the automated anatomic labeling tool implemented in bspmview*.

Next, we analyzed the correlation of subjective value (that is, WTP) with brain activity (GLM 2). As shown in previous studies, the vmPFC plays an important role in computing the subjective value across different modalities (Plassmann et al., 2007; Rangel et al., 2008; Hare et al., 2011; Bartra et al., 2013; Rangel, 2013; Clithero and Rangel, 2014). We therefore chose the vmPFC as a region of interest. The VS shows robust activation in GLM 1, and was also reported to be activated in a prior study on organic labeling (Linder et al., 2010); therefore, the VS served as a second region of interest. In GLM 2, we find that voxels in the vmPFC correlate with WTP (*p* < 0.05, SV FWE corrected), while the VS fails to reach this threshold (*p* = 0.08, SV FWE corrected, see Table 2.1). Further, we investigated the effect of FT labeling over and above what is already explained by WTP. Again, we find significant activation in the ACC, PCC and occipital lobe (*p* < 0.05, cluster-level FWE corrected). We also find significant striatal activity (*p* < 0.05, SV FWE corrected), see Table 2.2. Within a 10 mm sphere around the peak coordinate of the ventral striatum reported in Bartra et al. (2013), the cluster contains 70 suprathreshold voxels in GLM 1 and 27 suprathreshold voxels in GLM 2.

**Table 2:** **Parametric modulation analyses (GLM 2)**.

**Region name**	**Extent**	***t*-value**	***x***	***y***	***z***
**2.1. PARAMETRIC MODULATION WITH WTP**					
L Lingual gyrus	2312	8.09	−6	−76	−2
R Calcarine gyrus	2312	6.20	15	−82	10
L Fusiform gyrus	2312	4.43	−36	−64	−8
Small volume correction (VS)	6	3.24, *p* = 0.08	−3	11	−5
Small volume correction (vmPFC)	20	4.79, *p* = 0.03	−3	41	−14
**2.2. CATEGORICAL MODULATION WITH LABEL**					
L Cerebelum (VI)	5370	13.18	−15	−73	−8
R Lingual gyrus	5370	9.25	12	−79	1
L Middle temporal gyrus	5370	7.72	−45	−70	13
L ACC	186	4.24	−3	29	19
L MCC	186	3.43	−6	11	37
L Superior medial gyrus	186	3.39	−15	47	13
Small volume correction (VS)	32	3.52, *p* = 0.01	−9	17	−8

*2.1.: Parametric modulation with WTP at the time of stimulus onset. 2.2.: Categorical modulation with label at stimulus onset (Dummy regressor: 0 for conventional, 1 for FT products), both at p < 0.005. Only clusters that survive cluster-wise FWE correction (p < 0.05) are displayed. Also, small-volume correction results around the vmPFC and ventral striatum (10 mm sphere around sphere reported in Bartra et al., 2013) are shown. Brain regions are labeled according to the automated anatomic labeling tool implemented in bspmview*.

**Figure 4:**
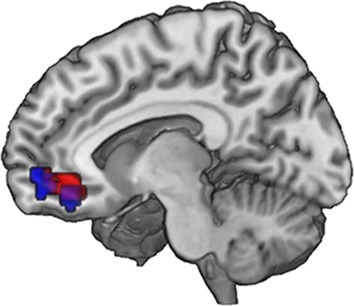
**Neural correlates of subjective valuation: In the ventromedial prefrontal cortex, overlapping voxels correlate with WTP across all pictures (GLM 2, red) and with the WTP difference (increment value) when evaluating an FT image (GLM 3, blue)**. Data are shown using MRIcroGL (MNI template) and in radiological convention.

**Figure 5:**
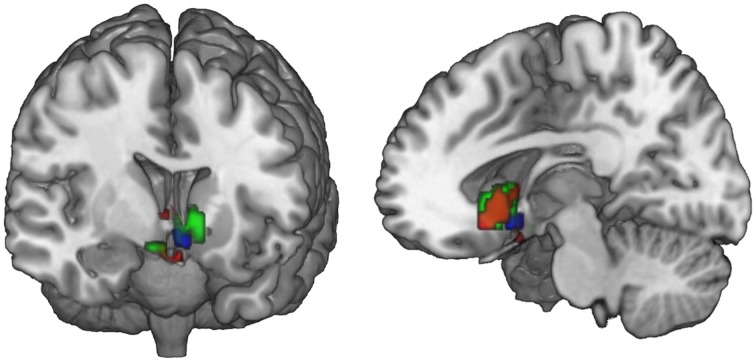
**Ventral striatal subregions responding value-independently and value-dependently**. In the ventral striatum, voxels that are active in response to the FT product (that may be due to salience or general responses to an FT label) are distinct from value-dependent voxels that correlate with the increment value of the FT product. In green, voxels are depicted that are activated in response to the FT vs. conventional product (GLM 1). The red voxels depict the activation in response to the FT product over and above what is already explained by WTP (GLM 2). The blue voxels correlate with the increment value of the FT product at the time of FT product presentation (GLM 3). In sum, a broader and more lateral part of the striatum is activated value-independently (GLM 2, green), and a smaller and more medial and ventral part is activated value-dependently (GLM 3, blue). Data are shown using MRIcroGL (MNI template) and in radiological convention.

Last, we correlated the increment value *i*, that is, the individual difference between an FT and the same conventional product, with the onset of all FT products in GLM 3. Both the vmPFC and the VS correlate with the increment value *i* (*p* < 0.05, SV FWE corrected) at the time of FT product presentation, see Table 3 and Figure 4 to see the overlap in vmPFC activity for correlations with WTP and increment value *i*. Importantly, the VS, voxels that correlate with *i* are distinct from those that are active in response to the label (GLM 1 and 2), see Figure 5.

**Table 3:** **Increment value of Fair Trade (GLM 3)**.

**Region name**	**Extent**	***t*-value**	***x***	***y***	***z***
R Middle frontal gyrus	33	4.75	51	38	16
L Rectal gyrus	59	4.26	−6	38	−14
L Mid orbital gyrus	59	3.13	−3	62	−8
L Calcarine gyrus	11	3.88	−3	−88	1
L Superior frontal gyrus	10	3.71	−18	29	46
R Temporal pole	13	3.70	27	5	−17
L Olfactory cortex	18	3.73	−3	11	−5
R Cuneus	11	3.23	9	−91	31
Small volume correction (VS)	10	3.73, *p* = 0.05	−3	11	−5
Small volume correction (vmPFC)	20	3.54, *p* = 0.03	−9	47	−8

*The table shows regions correlating with the increment value (that is, the WTP difference between Fair and conventional products) at the time of Fair Trade onset at p < 0.005. None of the clusters survive cluster-wise FWE correction (p < 0.05), therefore, uncorrected clusters are reported. Also, small-volume correction results around the vmPFC and ventral striatum (10 mm sphere around sphere reported in Bartra et al., 2013) are shown. Brain regions are labeled according to the automated anatomic labeling tool implemented in bspmview*.

#### PPI analyses

**Figure 6:**
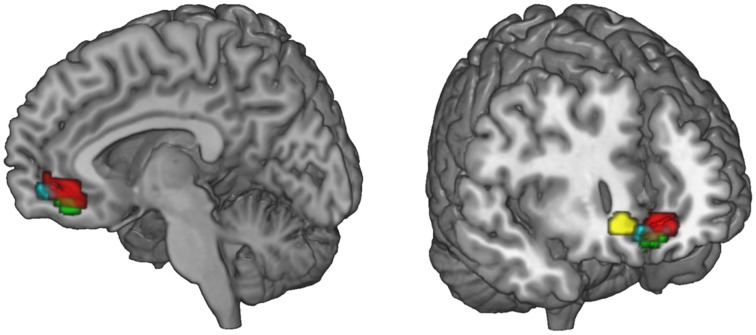
**Summary of the functional connectivity results**. WTP is correlated with voxels in the vmPFC (red, parametric modulation analysis, GLM 2). As WTP is higher for FT products, it is of interest to find out which regions exhibit increased task-related (FT vs. conv) functional connectivity with the vmPFC. We find that the superior frontal gyrus (green) and the ventral striatum (cyan) exhibit increased task-related connectivity in response to FT labels with the vmPFC (*p* < 0.05, small-volume FWE corrected). The ACC also exhibits increased task-related connectivity, but with a more lateral portion of the vmPFC that is not shown in the left slice. Data are shown using MRIcroGL (MNI template) and in radiological convention.

To investigate the network that may be responsible for the bias toward FT products, we performed PPI analyses. We were interested, whether the presence of an FT emblem (vs. no emblem) differentially modulates activity in the vmPFC, which correlates with the subjective value, or the VS, which was also shown to integrate different attributes (Haber, 2011). Seed regions of interest included the PCC, ACC, VS, and the SFG. The SFG shows robust activation in the contrast fair > conv and also correlates with WTP (at a more liberal threshold of p_uncorrected_ < 0.001). This region was also active in a prior organic labeling study (Linder et al., 2010). We were interested if these regions show increased task-related (fair > conv) coupling with the vmPFC, and possibly with the VS. These clusters may be critical in mediating the bias toward FT products, and increased WTP associated with FT products. We find that the VS, ACC as well as the SFG exhibit task-related increased functional connectivity with the vmPFC (*p* < 0.05, SV FWE corrected). The PCC does not show any task-related functional connectivity with the vmPFC. Neither the PCC, nor ACC and SFG show increased task-related connectivity with the VS. See Figure 6 for an overview and Table 4 for complete results.

**Table 4:** **Psychophysiological interaction (PPI) analyses**.

**Region name**	**Extent**	***t*-value**	***x***	***y***	***z***
**VENTRAL STRIATUM SEED**					
R Lingual gyrus	3956	12.09	12	−85	−2
L Fusiform gyrus	3956	10.07	−24	−58	−8
L Middle occipital gyrus	3956	9.31	−24	−79	19
Small volume correction (vmPFC)	13	3.74, *p* = 0.05	3	41	−14
**SUPERIOR FRONTAL GYRUS SEED**					
L Lingual gyrus	2480	7.12	−18	−76	−2
R Calcarine gyrus	2480	6.92	12	−91	10
L Middle occipital gyrus	2480	6.79	−18	−97	19
Small volume correction (vmPFC)	8	3.75, *p* = 0.04^*^	12	50	−8
Small volume correction (VS)	None				
**ANTERIOR CINGULATE SEED**					
L Lingual gyrus	3346	7.35	−12	−55	4
R Calcarine gyrus	3346	7.06	21	−85	16
L Calcarine gyrus	3346	6.42	0	−85	1
L Precentral gyrus	120	5.40	−45	−7	34
Small Volume correction (vmPFC)	9	3.34, *p* = 0.05	6	44	−14
Small volume correction (VS)	None				
**POSTERIOR CINGULATE SEED**					
R Middle occipital gyrus	2917	9.67	27	−85	19
L Superior occipital gyrus	2917	9.31	−15	−97	13
R Calcarine gyrus	2917	8.85	9	−91	7
Small volume correction (vmPFC)	None				
Small volume correction (VS)	None				

*The table shows regions showing increased task-related (task = onset Fair Trade pictures) with the seeds specified in the table at p < 0.005. Only clusters that survive cluster-wise FWE correction (p < 0.05) are displayed. Also, small-volume correction results around the vmPFC and ventral striatum (10 mm sphere around sphere reported in Bartra et al., 2013) are shown. Brain regions are labeled according to the automated anatomic labeling tool implemented in bspmview*.

* *Only survives voxel-wise FWE-correction*.

#### DCM

We find that there is a highly probable, rather small (directed) modulation of the vmPFC by all three regions. Please see the complete results in Table S1.

### Behavioral tasting task

Expected taste intensity, but not expected taste pleasantness, was significantly higher for FT products. Experienced taste intensity and pleasantness were both significantly higher for FT products, see Table 5. Expected and experienced taste intensity and pleasantness were significantly correlated only for conventional products, see Table 6.

**Table 5:** **Results of the behavioral tasting task**.

	**Median FT**	**Median conventional**	**Z**	***p*-value**	**Effect size *r***
Expected taste intensity	6	5	−**2.58**	**0.01**	**0.42**
Expected taste pleasantness	6	5	−1.66	0.097	0.27
Experienced taste intensity	5.5	5	−**3.16**	**0.002**	**0.51**
Experienced taste pleasantness	6	5	−**2.84**	**0.005**	**0.46**

*Ratings were analyzed using the Wilcoxon Signed-Ranks test and effect sizes r were calculated by r = $\left|Z\sqrt{N}\right|$ with N being the number of subjects used in the analysis, i.e., 38, and Z being the value of the test statistic. Bold values indicate statistically significant results*.

**Table 6:** **Correlation between expected and experienced taste pleasantness and intensity of identical chocolates**.

**Expected taste correlated with experienced taste**	**Correlation coefficient**	***p*-value**
Fair Trade intensity	0.171	0.235
Fair Trade pleasantness	0.237	0.101
Conventional intensity	**0.333**	**0.016**
Conventional pleasantness	**0.347**	**0.013**

*Correlation analyses of non-parametric data (Kendall's Tau correlation coefficient) between expected and experienced taste intensity and pleasantness. Bold values indicate statistically significant results*.

## Discussion

The present study investigated the neural and behavioral processes underlying the influence of FT labeling on food valuation and choice. Behaviorally, WTP was significantly higher for FT products. On the neural level, FT labeling increased activity in the VS, a region well-known to be important for reward processing, and also in the SFG, ACC, and PCC. Activity in the vmPFC correlated with subjective value, i.e., WTP. Parts of the VS and vmPFC also correlate with the increment value of FT products over conventional products at the time of FT product presentation. We also analyzed the network that may be responsible for the bias toward FT products and find that the SFG, VS, and ACC show increased task-related coupling with the vmPFC. Therefore, reward- and salience-induced activations may mediate the bias toward FT products by altering the input into the vmPFC. An additional chocolate tasting experiment with identical chocolates revealed a significant taste-placebo effect, i.e., experienced taste pleasantness and taste intensity were rated higher for FT labeled chocolates.

FT products were valued higher, in that WTP was significantly higher for FT compared to conventional products. This is in line with previous evidence that showed that consumer's natural and ethical concerns are important drivers for food choices, that the demand for FT products is rising, and that consumers are willing to pay more for FT products (Honkanen et al., 2006; de Ferran and Grunert, 2007). On the neural level, we find that the vmPFC is correlated with stimulus value, i.e., WTP. This is in line with previous studies that show a correlation between vmPFC activity and the subjective value of choice options (Plassmann et al., 2007; Hare et al., 2011; Bartra et al., 2013; Rangel, 2013). Also, activity in the vmPFC and the VS correlate with the increment value of FT products over the same products without an FT emblem, that is, the individual difference in WTP for FT compared to conventional products at the time of FT product presentation. This suggests that the striatal activity is not merely due to salience, but that part of the striatum also encodes subjective value differences. However, the difference in WTP is likely dependent on the original value of the product, therefore we cannot ascertain a true correlation between WTP and reward signal increases due to FT labeling.

Secondly, several regions show increased activation in response to the FT products, compared to conventional products. We expect that the activation in the occipital lobe for the contrast fair > conv is mainly due to additional visual input because of the FT emblem. Also, we find increased activation in the PCC, ACC, VS, and SFG. The PCC is a highly interconnected and metabolically active region (Leech and Sharp, 2014). Although the role of PCC in value-based decision making was largely unattended, recent meta-analyses showed that it correlates with stimulus values across tasks and reward modalities (Bartra et al., 2013; Clithero and Rangel, 2014). The PCC has been shown to be involved in coding reward outcomes, reward expectation and the encoding of delayed rewards (McCoy et al., 2003; Kable and Glimcher, 2007; Vassena et al., 2014). It is a key node of the default mode network and involved in learning, change detection, reward and task engagement (Pearson et al., 2011; Leech and Sharp, 2014). It is reciprocally connected with areas involved in attention and motivation, including the ACC, orbitofrontal cortex and caudate nucleus (Pearson et al., 2011). PCC neurons encode reward size and also respond to the omission of rewards and its variance, suggesting that this regions has a role in flexibly adjusting behavior (McCoy et al., 2003; Pearson et al., 2011). More complex behaviors, such as moral judgments, emotion and social cognition, are also thought to be processed in the PCC, among other regions (Greene et al., 2001, 2004). Another study found that the PCC/precuneus shows greater activity when thinking about duties and obligations, suggesting that the PCC is associated with a more outward-directed, social or contextual focus (Johnson et al., 2006). Heilbronner and colleagues found that decision salience signals are coded in the PCC. In contrast to other studies, they demonstrate that neurons in the PCC track decision salience, that is, the degree to which an option differs from standard, but not the subjective value of a decision (Heilbronner et al., 2011). Evidence also suggests that the PCC plays a role in regulating the focus of attention (Hahn et al., 2007; Leech and Sharp, 2014). We find that part of the PCC also correlates with WTP at a more liberal, cluster-uncorrected threshold of p_uncorrected_ < 0.005 (peak 1 at *x* = −3, *y* = −25, *z* = 31, *k* = 48, *t* = 3.44; peak 2 at *x* = −2, *z* = −49, *z* = 25, *k* = 13, *t* = 2.7). Although this correlation needs to be interpreted with caution due to the liberal threshold, it is in line with previous studies (Bartra et al., 2013; Clithero and Rangel, 2014). We conclude that part of the PCC is activated value-dependently, whereas another part generally responds to the FT label. This may be due because the FT option differs from standard (Heilbronner et al., 2011), or because of moral or social considerations (Greene et al., 2001, 2004; Johnson et al., 2006). Also, the FT label may be a signal for an environmental cue that implies behavioral change (Leech et al., 2012). However, our design does not allow us to distinguish between the different interpretations.

Further, the ACC showed robust activation in the main contrast fair > conv. The ACC has been associated with encoding values (Bartra et al., 2013; Clithero and Rangel, 2014). The ACC has strong connections to dopaminergic midbrain structure, and is thought to be involved in guiding behavior toward appetitive rewards (Hickey et al., 2010). It is critical for processing feedback from choices and inducing behavior change (Behrens et al., 2007; Walton et al., 2007; Quilodran et al., 2008). It is thought to monitor performance, and acts via prefrontal regions (MacDonald et al., 2000; Botvinick et al., 2001; Kerns et al., 2004) and also directly regulates behavior (Roelofs et al., 2006). It has been demonstrated that the ACC has a role in using reinforcement information to guide future behavior (Kennerley et al., 2006). Critically, the ACC, together with the anterior insula, is also part of the saliency network, that functions to segregate relevant internal and external stimuli in order to guide future behavior and select actions (Seeley et al., 2007; Menon and Uddin, 2010; White et al., 2010). The ACC plays a role in response selection (Bush et al., 1999). Single-neuron recordings showed that some of the ACC neurons are modulated by attentional demands (Davis et al., 2000). A study in monkeys found that neurons in the ACC do not encode the value of individual offers, suggesting that value comparisons take place upstream of the ACC (Cai and Padoa-Schioppa, 2012). In our study, we find that the ACC is activated independent of the subjective value of the options, as it does not correlate with WTP. The increased activity may be due to salience of the FT label. Also, it is conceivable that the label triggers cognitive processes, that are common for all FT products, but that do not vary for individual products. Such processes may include moral decision-making (Greene et al., 2001, 2004), or general reinforcement information (Kennerley et al., 2006). However, the exact mechanism needs to be clarified in future studies.

Quite unexpectedly, we find that the SFG is robustly activated in the main contrast fair > conv, correlates with WTP (at p_uncorrected_ < 0.001) and shows increased functional connectivity to the vmPFC. The SFG is located in Brodman area 10 and part of the frontal pole. In a previous study, it was also activated in response to organic labeling (Linder et al., 2010). It has been shown to be important for planning and executing highly abstract goals and response strategies (Coutlee and Huettel, 2012). This region was also shown to be more active in moral scenarios compared to non-moral scenarios (Greene and Haidt, 2002), and when thinking about personal intentions and consequential actions (den Ouden et al., 2005). Studies showed that this region is active during exploratory decisions, that is, choosing unfamiliar options that may turn out to be more advantageous and improve future decisions, compared to exploitative decisions, that is, deciding on accumulated experience (Daw et al., 2006). Another study found that, while the vmPFC encodes the relative value of a current decision, the frontal pole may promote rather long-term behavioral flexibility during voluntary choice by tracking the relative advantage in favor of switching to alternatives. The frontal pole was shown to be active when a change in behavior occurs, and that it continually tracks the long-term evidence to adapt behavior (Boorman et al., 2009). Other studies suggest that it serves to hold in mind goals while exploring sub-goals (Koechlin et al., 1999). It was shown to be important for cognitive branching based on reward expectations and integrating outcomes of multiple conjectures (Koechlin and Hyafil, 2007). It is conceivable that the SFG shows higher activation in response to FT labels due to its role in diverging attention across different goals and tracking advantages of possibly unfamiliar options.

Also, the VS shows increased activation in response to food items labeled with an FT emblem. Originally, the VS was shown to be associated with approach and avoidance motivation for preparation of motor responses (Salamone, 1994; Schultz, 2006). The VS is the “epicenter” of dopaminergic neurons and therefore involved in motor response initiation (Mogenson et al., 1980; Schultz, 2006; Aupperle and Paulus, 2010). It was also consistently shown to be important for reward processing and directing decisions to the current optimum (Olds and Milner, 1954; O'Doherty et al., 2002; Schultz, 2006; Aupperle and Paulus, 2010). The VS signals reward values of outcomes (Schultz, 2006; Floresco et al., 2008; Aupperle and Paulus, 2010) and is important for adapting, i.e., learning new behaviors (Aupperle and Paulus, 2010; Haber, 2011). While many rewards are necessary for survival, humans also respond to rewards such as money, power or visual beauty (Schultz, 2006). For example, objects signaling wealth or social dominance were shown to act as strong social reinforcers modulating the dopaminergic reward circuitry, revealed by increased activation in the VS, among others (Erk et al., 2002). A previous study on the neural correlates of processing product labels, in this case, a low-fat label, reduced the rewarding properties of food products, and decreased the food's appeal (Ng et al., 2011). In contrast to that, organic labeling increased the food's valuation on the behavioral and neural level (Linder et al., 2010). Critically, we also investigate the effect of FT labeling over what is already explained by differences in WTP. GLM 2 shows less, but significant striatal activity. Activity in these voxels is therefore probably value-independent and may be due to salience, among others (Zink et al., 2003). Zink and colleagues found increased VS activity after infrequent, and therefore more salient, distractor occurrences. Critically, a subregion of the VS (the caudate, in contrast to the nucleus accumbens) was activated only when the distractors were behaviorally relevant, that is, the distractors required a response (Zink et al., 2003). Here, we find that a broader, more lateral part of the VS is activated value-independently. A smaller and more ventral and medial portion of the VS correlates with the increment value of FT products at the time of FT product presentation. This is in line with previous findings that show that the VS is a rather diverse region, with input terminating in distinct sub-regions. For example, afferents from the vmPFC terminate within the shell and in the medial wall of the caudate nucleus, whereas input from the anterior cingulate cortex, for instance, terminate mainly in the more lateral-dorsal parts of the ventral striatum (Haber, 2011). However, future studies are necessary to ascertain a direct link between striatal activity and reward signaling in response to social sustainability labeling.

Previous studies, as well as our study, suggest that labeling can influence valuation of food products, as seen by increased WTP and indirectly via vmPFC activity. It is therefore of interest to analyze the network underlying the change in valuation in favor of FT products. Therefore, we conducted several PPIs to analyze task-dependent changes in connectivity between regions active in the fair > conv contrast, and the vmPFC region of interest. We find increased coupling between the SFG and VS with the vmPFC when an FT emblem was presented, compared to no labeling. Also, the ACC shows positive connectivity with a more lateral part of the vmPFC. The vmPFC and VS are densely connected (Hedreen and Delong, 1991; Haber et al., 1995; Goldstein and Volkow, 2002; Haber, 2011; Yu et al., 2013), and those connections are important for e.g., reward-based learning (Haber et al., 2006). None of the regions show increased task-related connectivity with the VS, which would argue against striatal integration of reward information (Haber et al., 2006; Haber, 2011) in our study. The increased functional connectivity between the vmPFC and the other regions in response to an FT emblem may be unidirectional or bidirectional. However, this cannot be fully tested using functional connectivity analyses. For example, efferent projections from the VS project primarily to subcortical regions (Haber et al., 1990), the VS can also influence the cortex directly as axons directly project to the basal forebrain, which is the major source of cholinergic fibers projecting to the cerebral cortex (Beach et al., 1987; Chang et al., 1987; Haber et al., 1990; Záborszky and Cullinan, 1992; Haber, 2011). Therefore, we also conducted a DCM analysis using a rather novel routine (DCM *post-hoc*, (Rosa et al., 2012)). We find a highly probable directed modulation of the vmPFC by the three regions identified in the PPI analyses. The rather small strength may be due to the fact that we proceeded with the DCM routine with fewer participants (*N* = 19). The DCM analysis corroborates the hypothesis that the vmPFC activity is modulated by the other regions, and not vice versa, suggesting a network that alters the valuation of the products.

In the behavioral tasting task, we find increased experienced taste pleasantness and intensity for the FT labeled—but identical—chocolate. Several different marketing actions have been shown to induce so-called marketing placebo effects, i.e., modifying contextual components of a product can alter experienced pleasantness and efficacy of identically composed products (Shiv et al., 2005; Grabenhorst et al., 2008; Plassmann et al., 2008; Plassmann and Weber, 2015). For instance, taste perception was shown to be affected by knowledge about a product's brand, its ingredients or packaging (Allison and Uhl, 1964; Lee et al., 2006). Also, a priori expectancies about a product can alter consumption experiences (Plassmann and Weber, 2015). We also found correlations between expected and experienced taste ratings; however, they were lower than expected, and not significant in the FT condition. Self-reported taste-placebo effects can also be seen on the neural level, showing that consumption experience can be altered by cognitive concepts (de Araujo et al., 2005; Plassmann et al., 2008; Plassmann and Weber, 2015). Morally loaded labels, such as FT and organic emblems, are marketing cues that attract consumers (Sörqvist et al., 2013). A previous study found that participants were willing to pay more for organic coffee, even when they preferred the taste of the non-labeled alternative. Further, organic coffee also led to a more favorable perceptual experience of the coffee, i.e., higher taste ratings. Critically, they ruled out social desirability effects by replicating this effect in anonymous participants (Sörqvist et al., 2013). Bratanova et al. (2015) found that products declared as coming from ethical production increased taste experience and led to higher willingness to pay for the respective products. They suggested that products of ethical origin increase moral satisfaction, which then enhances taste expectations and taste experience. The superior taste experience then reinforces future purchasing habits (Bratanova et al., 2015). They found that individual values moderated the link between a food's ethical origin and increased taste perception, i.e., only people who endorse the values conveyed by a product's name or label also experience greater moral satisfaction from consuming the product (Bratanova et al., 2015). However, only self-reports and hypothetical WTP was measured. Here, we report WTP from a real bidding task as well as neural measures of perceiving ethical labeled products. Another study suggests an explanation for the interaction between consumers' values and taste experience—consumers assess the taste of a food by comparing internalized value priorities and the values symbolized by the product; in case of congruency, consumer's attitudes are more positive and also taste experiences are better (Allen et al., 2008). For example, organic product choice is also highly correlated with individual attitudes about health and environmental consequences (Gunne Grankvist, 2001). Besides increases in taste preference, social ethics claims on food packages may also induce health halo effects, i.e., they promote misperceptions about the food's healthiness and calorie content, as subjects seem to extrapolate positive FT attributes, such as better social standards, also to health evaluations (Schuldt et al., 2012) and, as shown here, to better taste evaluations. However, future research is needed to clarify underlying neuronal processes, for example with a taste experiment within the fMRI scanner, as previously undertaken with low-fat labels and wines at different prices (Plassmann et al., 2008; Ng et al., 2011). A more detailed understanding of neural mechanisms underlying certified food product choice and consumption is important to understand marketing trends and to develop policy interventions with which food choice can be positively influenced (Plassmann and Weber, 2015), i.e., restricting specific label types and allowing them only on rather healthy products.

Certain limitations of this study need to be considered. As we compared a condition with an FT emblem vs. a no-label condition, a possible confound is the colored FT emblem, as it is visually more salient. We opted for this design because this resembles a real-world situation, i.e., conventionally produced products do not carry a label. Indeed, Zink and colleagues have shown that salience increases activations in the VS independent of value (Zink et al., 2003). A previous study used a gray non-organic emblem vs. a colored organic emblem and also found increased striatal activity (Linder et al., 2010). We find that part of the VS correlates with the increment value for FT products at the time of FT product presentation. This renders complete value-independent VS activity rather unlikely. Also, the fMRI task, and mainly the behavioral tasting task, may have induced a social desirability effect. However, we believe that neural measures, especially subcortical activations as seen in the VS, are more difficult to manipulate consciously. Further, a previous study showed that taste placebo effects of organic products are comparable when subjects remain anonymous (Sörqvist et al., 2013), however, we cannot fully rule out this effect. Products were shown twice—once with a FT label and once without, and this may have influenced the participants. Subjects were told that they would receive products in the end, depending on their bids—and they could receive the conventional or the FT version of the products. The products they saw inside the scanner were therefore rather “symbolic” for the kind of product they would receive in the end. Further, if subjects grew suspicious due to the fact that they saw every product twice, we believe that we would not have observed the strong placebo effects in the subsequent behavioral tasting task.

A computational model of value-based decision making in the food domain suggested that food items can be sub-divided into different attributes, and a value is assigned to each of the attributes. In addition to very basic attributes of a food product, such as nutritional content and taste, also abstract attributes are taken into account. However, those abstract attributes vary between individuals, and can comprise for example health consequences (Rangel, 2013). Social sustainability claims on products are rather abstract product attributes that may be taken into account by some individuals. The FT emblem may be more salient, and guide attention toward abstract product attributes. Alternatively, the label may be more rewarding itself, or elicit complex cognitive processes. Taken together, our study demonstrates that FT labeling of food products has a variety of effects on behavioral and neural measures. On a behavioral level, FT labeling induces a so-called taste-placebo effect and, secondly, increases WTP in a bidding task. Also, FT labeling increases activity in the VS, a region well-known for its role in reward-processing, as well as in the ACC, SFG, and PCC. We find increased task-related connectivity between the VS, ACC, and SFG with the vmPFC. We suggest that these regions, which are important for reward-related processing, salience and possibly higher cognitive functioning, influence value computations in the vmPFC. Our study provides evidence for a potential neural mechanism that mediates effects of social sustainability signals, and may improve future public policy interventions that aim at improving consumer choices.

### Conflict of interest statement

The authors declare that the research was conducted in the absence of any commercial or financial relationships that could be construed as a potential conflict of interest.
