# Supplementary material for: Effects of social sustainability signaling on neural valuation signals and taste-experience of food products
Source: Front Behav Neurosci. 2015 Sep 8;9:247. doi: 10.3389/fnbeh.2015.00247 (PMC4561672; doi:10.3389/fnbeh.2015.00247)
Supplement: Supplementary file 1 [file DataSheet1.DOCX]

**Supplementary Material**

**Dynamic causal modeling**

We further investigated the effective connectivity between the regions of interest that show a significant PPI effect with the ventromedial prefrontal cortex (vmPFC), that is, modulation by label between the region of interest and the vmPFC.

Therefore, we used a relatively novel procedure, a “post-hoc” dynamic causal model (DCM) selection (Rosa et al., 2012) to find the best model out of all possible connection structures. Using this procedure, we conducted an exhaustive search for the best of all models, as well as family-level inference about connection and modulation strengths (Rosa et al., 2012; Friston and Penny, 2011). Conventional DCM analyses with several nodes and only few prior hypotheses about the nature of the network lead to a combinatorial mass of all plausible models, that makes it computationally expensive to estimate all possible models in model space (Hillebrandt et al., 2013). DCM was performed by following the steps (detailed below):

a. extracting of BOLD fMRI time series from each individual using individual coordinates

b. Specification of model space based on a fully, bi-directionally connected model and subsequent estimation of the model

c. Post-hoc model selection

DCM analyses were performed with DCM12 implemented in SPM 12 (in contrast to the other analyses, which were performed in SPM 8. This is due to the fact that the new release provides an improved algorithm and the hyperpriors were adjusted to reflect a more realistic signal-to-noise ratio in the VOIs (Hillebrandt et al., 2013)). Regions of interest included the anterior cingulate cortex (ACC), ventral striatum (VS), superior frontal gyrus (SFG) and vmPFC.

1. VOI extraction

First, we extracted all volumes of interest (VOIs) based on individual peaks of the Fair > conventional contrast using the SPM 12 Eigenvariate toolbox. For the DCM analysis, we extracted each participant’s principal eigenvariate around the individual-specific local maxima activation nearest to the peak voxel of the second level group analyses. The radius of the VOI spheres was 8 mm, and the search radius for local maxima from the group analyses was restricted to 12 mm for all regions, except for the VS, where we limited the search to 8 mm due to its smaller size. All voxels were significant at p<0.1 uncorrected with an extent threshold of at least 5 voxels. The time series were adjusted for effects of interest. After applying these somewhat stricter criteria (compared to the PPI analysis), we had to exclude 11 participants because they failed to show any supra-threshold voxels in one or more of the nodes, or because we could not extract more than five voxels.

1. Specification of the fully connected model

For running the DCM analysis, we created and estimated a new GLM, with the onset of all pictures as a first regressor, and the contrast FT > conv as a categorical modulator (by using a dummy regressor as a modulator, with -1 for conventional and +1 for FT products), as well as nuisance regressors (bidding period and movement). The first regressor then served as driving input, whereas the contrast was our modulatory input. We created and estimated a fully-connected DCM. This was a “full model” in a sense that it incorporated all reciprocal fixed connections between and within the VOIs of interest (as specified in the DCM.A matrix). To simplify the models and for better interpretation, we did not allow the modulation of self-connections within each region (Hillebrandt et al., 2013), by setting the diagonal in the DCM.B matrix (which describes the modulation of coupling strength between brain regions as a result of a task condition) to zero. The driving input was allowed to enter any node (DCM.C matrix). The DCMs were deterministic (i.e., we did not model noise, as opposed to stochastic (Daunizeau et al., 2012), bilinear (as opposed to nonlinear, see (Stephan et al., 2008), one-state models (as opposed to two-state models, (Marreiros et al., 2008) and we used mean-centered inputs. Three additional participants were excluded as the model did not explain sufficient variance (i.e., less than 3%).

1. Post-hoc model selection

To explore all possible DCMs, a post-hoc model selection routine was applied for the remaining 19 participants (Friston and Penny, 2011). The search takes a subset of parameters with the least evidence, and searches over all reduced models within that subset (by turning connections “off”). It is noteworthy that with more than 8 parameters, the post-hoc routine implements a “greedy search” over all models formed by removing all permutations of eight parameters whose individual removal produces the smallest reduction in model evidence, resulting in 2^8^ reduced models. All possible combinations of disabling these parameters are evaluated, the script then uses the model with the greatest evidence, and repeats the steps until no more connections can be pruned (Crone et al., 2015; Rosa et al., 2012; Friston and Penny, 2011). Critically, post-hoc routines were shown to yield very similar results, compared to conventional DCM approaches, while requiring much lower computational time (Rosa et al., 2012). Bayesian model selection (BMS) uses the “best model” approach to select a winning model with the highest evidence to make inferences based on the parameters of that model. However, in a large model space, this approach becomes rather brittle (Penny et al., 2010). In the post-hoc routine, the model space is very large, and therefore the posterior mass is diluted over a high number of models. As a consequence, for post-hoc DCM, one usually does not look at the winning model on its own, as usually no single model has a high probability. This is because all models that share some characteristics with the winning model will have a non-zero probability in the presence of noise (Rosa et al., 2012; Hillebrandt et al., 2013). Family level inferences allow to disambiguate the inputs to the system and the connections by estimating the posterior family probabilities from a fixed-effects analysis (Penny et al., 2010). The posterior probabilities (as saved in this routine) splits the model space into families of models and tests whether a family of models without a certain parameter has a higher probability than the family with this parameter (Penny et al., 2010). Bayesian parameter averaging (BPA) within families provides inferences of parameters that are independent of assumptions of model structure. We take 0.9 as strong evidence (the posterior probability of models containing connection X over models not containing this connection), and a lower value as a trend that this connection exists.

Results:

The winning model was model 256, with an evidence of 0.1. The next probable model had a posterior probability of 0.07. When dividing the probability of the winning model by the probability of the second most probable model, the Bayes factor is 1.4, which is not considered as positive evidence (Kass and Raftery, 1995). As mentioned above, in large model spaces like ours, it is to be expected that the posterior mass is diluted over a high numbers of models, as all models that share some characteristics with the full winning model will have a non-zero probability in the presence of any noise. Also, in synthetic data, Rosa and colleagues found similar results (Rosa et al., 2012; Hillebrandt et al., 2013). We report results from the Bayesian parameter averaging – family-level posterior probabilities (DCM.Pp) as well as parameter estimates obtained from the winning model (DCM.Ep). We find that there is a highly probable, rather small (directed) modulation of the vmPFC by all three regions, thereby replicating the PPI analyses, which, however, did not make any inferences on directionality. In contrast to PPI, the DCM analysis also makes inferences on directionality, and the results suggest that the vmPFC activity is indeed modulated by the regions of interest. In addition, the vmPFC also negatively modulates ACC activity. Please see the complete results in table S1.

**Table S1: Estimated DCM coupling parameters**. The table shows coupling parameter estimates of the winning model and posterior probabilities (in parentheses). Posterior probabilities (over models) are from family comparisons, comparing a family that has this connection with a family that does not. Probabilities and coupling parameters are rounded to two decimal places and those parameters whose probability is equal or greater than 90% are shown in bold. Driving input enters ACC **(-0.02 (1)**) and SFG (**0.09 (1)**)**.** If the numbers are in italics, this value was set to zero at the time of model specification of the full model. Columns = coming from, rows = coming into. In SPM12, the parameterization of the self-connections (the leading diagonal of the A-matrix) are log-scaled and the prior expectation is fixed at -0.5. Consequently, a positive value means that the self-connection is more inhibitory and a negative value means that it is less inhibitory compared to its prior.

|  | ACC ⇒ | SFG ⇒ | vmPFC ⇒ | VS ⇒ |
| --- | --- | --- | --- | --- |
| FIXED COUPLING |  |  |  |  |
| ACC | -0.79 (0.81) | **0.04 (1)** | -0.09 (0.66) | **0.16 (1)** |
| SFG | **0.05 (1)** | -0.65 (0.85) | -0.12 (0.84) | 0.01 (0) |
| vmPFC | **0.19 (1)** | **-0.01 (1)** | -0.33 (0.68) | **0.29 (1)** |
| VS | **-0.17 (1)** | -0.01 (0.85) | **0.06 (1)** | **-0.84 (1)** |
| MODULATION BY FT>Conv |  |  |  |  |
| ACC | *0* | **0.24 (1)** | **-0.16 (1)** | 0.26 (0.77) |
| SFG | **-1.05 (1)** | *0* | 0 (0) | **-0.53 (1)** |
| vmPFC | **0.29 (0.92)** | **0.05 (1)** | *0* | **0.01 (1)** |
| VS | **-1.15(1)** | **0.26 (1)** | 0(0) | *0* |
